# A study on the emotional and attitudinal behaviors of social media users under the sudden reopening policy of the Chinese government

**DOI:** 10.3389/fpubh.2023.1185928

**Published:** 2023-08-04

**Authors:** Qiaohe Zhang, Tianyue Niu, Jinhua Yang, Xiaochen Geng, Yinhuan Lin

**Affiliations:** ^1^Academy of Fine Arts, Huaibei Normal University, Huaibei, China; ^2^Academy of Arts & Design, Tsinghua University, Beijing, China; ^3^College of Humanities, Tongji University, Shanghai, China; ^4^School of Art, Nantong University, Nantong, China; ^5^Xiamen Academy of Arts and Design, Fuzhou University, Xiamen, China

**Keywords:** COVID-19, deregulation of epidemic control, emotional and attitudinal behaviors, social media users, artificial intelligence, natural language processing

## Abstract

**Introduction:**

Since the outbreak of the COVID-19 pandemic, the Chinese government has implemented a series of strict prevention and control policies to prevent the spread of the virus. Recently, the Chinese government suddenly changed its approach and lifted all prevention and control measures. This sudden change in policy is expected to lead to a widespread outbreak of COVID-19 in China, and the public and local governments are not adequately prepared for the unknown impact on society. The change in the “emergency” prevention and control policy provides a unique research perspective for this study.

**Methods:**

The purpose of this study is to analyze the public's attitudes and emotional responses to COVID-19 under the sudden opening policy, identify the key factors that contribute to these attitudes and emotions, and propose solutions. In response to this sudden situation, we conducted data mining on topics and discussions related to the opening of the epidemic on Sina Weibo, collecting 125,686 interactive comments. We used artificial intelligence technology to analyze the attitudes and emotions reflected in each data point, identify the key factors that contribute to these attitudes and emotions, explore the underlying reasons, and find corresponding solutions.

**Results:**

The results of the study show that in the face of the sudden release of the epidemic, the public mostly exhibited negative emotions and behaviors, with many people experiencing anxiety and panic. However, the gradual resumption of daily life and work has also led some people to exhibit positive attitudes.

**Conclusion:**

The significance of this study is to help the government and institutions understand the impact of policy implementation on users, and to enable them to adjust policies in a timely manner to respond to potential social risks. The government, emergency departments, and the public can all prepare for similar situations based on the conclusions of this study.

## 1. Introduction

As of September 20, 2022, the global outbreak of COVID-19 has caused 229 million cases of infection and 4.75 million deaths (1). The outbreak of the epidemic has the characteristics of suddenness, rapidity, and unpredictability. It often poses a serious threat to the life and health of the public and triggers complex emotions such as panic, dissatisfaction, and anger (2). Three years after the first COVID-19 case, China announced the end of restriction measures (later compared to the rest of the world) and experienced a large-scale spread of infections for the first time. The Chinese government announced the “Dynamic Zero COVID-19 Strategy” on January 23, 2020. On December 7, 2022, China suddenly promulgated the “The New 10 Measures” policy for epidemic prevention and control and announced the national release of the blockade (3). China's local government and the public were not prepared for the sudden change in epidemic policy. Due to the government blockade and the implementation of various security measures, the epidemic not only destroyed economic development but also changed people's lives during the pandemic. At the same time, it also has an important impact on people's mental health (4).

The public is easily influenced by various government information, and government policies are also changing. In the early days, the public obtained government information through traditional media such as newspapers and television, but lacked feedback channels. With the emergence of the Internet, the public can obtain relevant information faster, but the feedback speed is slow. Nowadays, in the era of mobile Internet, government information quickly spreads to the public (5), and the public can also make timely responses to government policies, forming a communication channel of rapid dissemination and feedback. In this case, social media has become a part of public life, and due to public concerns, the use of social media during the pandemic has become more frequent, which can not only maintain social distance but also obtain more information. The government's reputation has also shifted from eWOM to mWOM (6), and the public's feedback on government policy implementation has also changed accordingly. This is also the reason why social media users were chosen as the research object in this study, as they can provide timely data and reflect the real public attitudes.

A large amount of information that is difficult to distinguish between true and false is widely disseminated by social media, and its adverse effects are comparable to the virus epidemic, stimulating the public's nerves, stirring up social emotions, and creating extremely harmful social risks. The World Health Organization calls it “Information Epidemic”. The so-called information epidemic refers to the fact that at the same time the epidemic, excessive information (some are correct and some are wrong) makes it difficult for people to find trustworthy information sources and reliable guidance (7). In the context of the information epidemic, people have a collective panic because of various information related to the epidemic, and changes in mood and attitude have seriously affected the epidemic prevention and control and social stability.

Understanding the public's emotional attitude has practical significance for current and future public health management. The public's emotions and views on the risk of the epidemic determine their personal behavior and whether they cooperate with the necessary government control measures (7, 8). Mastering the public's emotional attitude and the influencing factors will help the government in implementing effective policies to address the psychological trauma and fear experienced by the public after the COVID-19 epidemic.

As a representative Chinese online social media platform (9), Weibo is designed to facilitate interaction between users. The interaction in the Weibo environment is related to users' emotions. The communication and social value transmission brought by the social interaction of online media will allow users to experience positive emotional value changes. The close interaction of microblog users has also brought huge user value (10), user emotion and user satisfaction, and is also the embodiment of user behavior in the online social media space.

In the research of network social media users, some studies believe that posting information through social media is a behavioral plan for educating users without understanding users' needs and problems (11). However, recent studies have shown that online social media has higher user engagement than other traditional media (12). With the popularity of web social media, the Chinese government has also extended the category of government policy information used to web social platforms such as Weibo to provide information services to users (13). In emergency response, the release of government policy information mainly considers the user's adoption of the information posted by the government's official social media, and studies have been conducted on the factors that affect users' adoption of government information. Some studies propose that user communication through social media, and the adoption of government policy messages, is a value manifestation of strong democratic countries (14). In a study of user behavior analysis, sentiment analysis of Twitter posted information on U.S. presidential candidates evaluates the link between user tweets and realistic elections (15). Studies of this type of user behavior have mainly focused on a small number of social media influencers' behavioral impacts. It also shows that users' emotional behavior is influenced by other users' online emotional expressions (16). Aspects of emotion impact research targeting group social users. When encountering a disaster event, extracting network social media user behaviors can help emergency responders form stronger situational awareness of the disaster area itself (17). In China, there is a strong correlation between online social user behavior and online political contention. At the individual level, the emotional response to public events not only shapes user interpretation but also significantly impacts the social behavior of network users and the construction of public discourse. From the social level, social culture not only influences users' emotional reactions but also determines the mode of government interactions with the public and the basic framework construction of Cyberpolitical contention (18).

In this context, behavioral studies of social media users provide a more comprehensive picture of the impact of the outbreak on social development. Although numerous studies have analyzed users of social media in the face of a “post-epidemic” era, especially when the Chinese government suddenly announced a release of epidemic policy (the epidemic did not end but rather expanded further). The information of social media users is characterized by timeliness, and the research on its behavior is a key issue in this phase of this pandemic, helping the government to respond to public opinion pressure and adjust related policies in a timely manner, meanwhile, there is also relatively little research in this area.

Emotion and attitude are parts of user behavior. In the research of user's emotional attitude, the comments with obvious emotional color and emotional tendency published by users have an important impact on enterprise performance (19, 20), public policy (21), and political decision-making (22). In recent years, user emotional analysis has emerged as a novel research approach in various fields, including entrepreneurial success (23), environmental factors in the tourism industry (24), and political decision-making and planning (25). Similarly, this has led to government departments and social organizations needing to obtain effective social feedback by analyzing and mining user emotional data, providing reliable research basis for government political decision-making and planning. Sentiment analysis on online social media extracts valuable information from natural language text to provide decision-makers with structured and actionable knowledge (26). In the process of spreading user emotions, emotional user comments are forwarded more frequently and faster than neutral online user comments (27). From the perspective of emotional media communication, there is a difference between the way users communicate their emotions through online media and face-to-face communication, and the impact of information transmitted through online media on the recipient's emotions is different from face-to-face communication. The spread of emotion in the network is mainly judged by online comments. Chinese-related online reviews are user-oriented, have a wide range of influence, and can be measured (28). The machine learning method is used to extract the emotion in online comments, and the Markov blanket model is introduced to capture the emotion in the text through conditional dependence between words and keywords and high-frequency words (29). In-group comments are usually carried out through a topic, and online comments on Weibo tend to focus on its relevant “topic”. The “topic” in social media will affect user behavior (30). In view of the concentration and short text characteristics of Chinese microblogging topics in this study, Chinese microblogging is realized through topic clustering and emotional analysis, as well as prediction of hot issues in microblogging and emotional analysis of user comments (31).

Users who rely on social media for COVID-19 information may experience increased anxiety symptoms and decreased trust in the information. However, this reliance does not significantly affect preventive behavior, leading to citizens confusion regarding the adoption of preventive measures. More research is needed in this field since limited studies have explored the impact of anxiety on attitudes and behavior during the COVID19 pandemic (32). Uncertainty is a common emotional response during a health crisis, such as the COVID-19 pandemic, as it arises from perceiving an invisible threat with unpredictable outcomes. Effective communication of health information can help alleviate the fear induced by uncertainty (33).

The most direct manifestation of reflection on epidemic prevention and control policies is emotional expression (34). Through the analysis of emotions, we can understand the impact of relevant policies on society. In this situation, tracking, identifying, and analyzing the evolution of public sentiment through the mining of massive social media data not only helps to identify public emotions and implement psychological counseling, but also provides computational support for grasping the trajectory of event development and improving emergency management effectiveness. Therefore, the emotional attitude in the context of information epidemics has become a new research field that is highly concerned by various sectors.

The current research aims to explore the emotional attitude of the Chinese public and its influencing factors after the opening of China's epidemic policy. A questionnaire survey is a common data collection method in this research direction (35), but due to the limited amount of data, it is difficult to grasp the research problem in a macro and in-depth way. Using the method of big data text mining, researchers can summarize and study the impact of epidemic policy on public sentiment and attitudes and the factors behind it. Text mining is a new field in user emotion research. It collects data through the network to observe human psychology and behavior (36, 37). At present, researchers have used this method to measure the public's reaction to government policies such as mental health and social prejudice (38, 39). In the aspect of emotional attitude research, we also began to use this method to carry out relevant research (40). The use of large samples in big data mining implies strong objectivity, high timeliness, and significant impact, making it a more suitable method for this study.

Due to the gradual alternation of restrictive and permissive measures, European and American public health policy research has primarily focused on assessing the effectiveness of adopted policies and their economic consequences, rather than sentiment analysis. In contrast, China government has recently triggered relevant and emotionally charged discussions on social networks, by shifting from 3 years of strict epidemic control policies to a sudden opening up policy announced within a few days (41). Previous Chinese sentiment research has predominantly examined the emotional impact of early-stage epidemic prevention and control measures (34, 42), with less emphasis on subsequent “changes” in prevention and control policies as the epidemic situation improves. This article aims to investigate the attitudes and emotional behaviors of social media users in response to the abrupt shift in epidemic prevention policies during the period of December 1st to December 20th, 2022. The primary research questions addressed are as follows:

1. What are the prevailing attitudes among social media users?
2. How do social media users express their emotions under different attitudes?
3. What factors influence these attitudes and emotions?

## 2. Related work

This section discusses related work in three parts: data acquisition, training of text classification models, and sentiment analysis based on media users.

### 2.1. Weibo data mining

Sina Weibo is one of the most popular social media applications in China. A significant amount of work has been done to study the data of Sina Weibo. For example, Samuel et al. (43) used Weibo data to analyze social media behavior and emotional changes during emergency events. Garcia and Berton (44) used the TPACK framework to explore the design and implementation of teaching by Chinese early childhood education workers during the epidemic period using Weibo data. Boon-Itt and Skunkan (45) studied people's attitudes toward wild animals on Weibo to analyze public opinions on stray cats in China. Naseem et al. (46) used Python technology to collect Weibo data containing “Shanghai” during the epidemic period to study the public's attitude toward the image of Shanghai during the COVID-19 pandemic.

### 2.2. Text classification model

With the explosive growth of information, the method of manually classifying data by humans has become outdated, and using machines to automate data annotation has significant significance. Currently, there are many deep learning-based methods for text classification. TextCNN (47) uses convolutional neural networks for text classification, and its network structure is relatively simple, so the number of network parameters is small, the calculation is small, and the training speed is fast. HAN (48) uses a hierarchical structure to not only calculate attention between words, but also calculate attention between sentences. When the text/document is long, it can still obtain relatively good classification results. FastText (49) uses the method of word vector to classify text, which originated from Google's work word2vec (50, 51). FastText's model is relatively simple, so its inference speed is fast, and its accuracy is also high. Subsequently, some text classification methods based on pre-trained models gradually became mainstream, and they only need a small amount of data to achieve very good classification effects when completing specific tasks.

### 2.3. Sentiment analysis

There is a large amount of work utilizing data on social media for sentiment analysis during the COVID-19 pandemic. Samuel et al. (43) collected COVID-19 related tweets and used naive Bayes and logistic regression classification methods for sentiment analysis. Garcia and Berton (44) used topic identification and sentiment analysis to study a large number of tweets from Brazil and the United States, two countries with high numbers of transmission and deaths during the COVID-19 pandemic, analyzing the long-term emotional trends and their relationship with published news. Boon-Itt and Skunkan (45) conducted data mining on Twitter, collecting a large number of tweets for keyword frequency analysis, emotion analysis, and topic modeling, using natural language processing methods and latent Dirichlet allocation algorithm to identify the most common Twitter topics. Naseem et al. (46) collected a large number of tweets related to the COVID-19 pandemic on Twitter, conducting a post-evaluation of the early information flow on social media during the COVID-19 pandemic, providing information for policies applicable to social platforms. The above work conducted sentiment analysis on social media users during the COVID-19 pandemic, but lacked sentiment analysis on social media users in the context of sudden changes in epidemic policies.

## 3. Method

We carried out a sentiment analysis conducted on the public response to the Chinese government's announcement of removing COVID-19 mobility restrictions. The analysis was based on the following steps:

1. Data collection: we acquired sample data for analysis.
2. Data pre-processing: we conducted data cleaning to improve quality of results and manual labeling to enhance the training process.
3. Data segmentation: we transformed unstructured text into structured data through segmentation.
4. Text classification: we assigned attitudes and emotions to each comment using text classifications technics.
5. Text analysis: we extracted underlying factors through text analysis methods.

**Figure 1:**
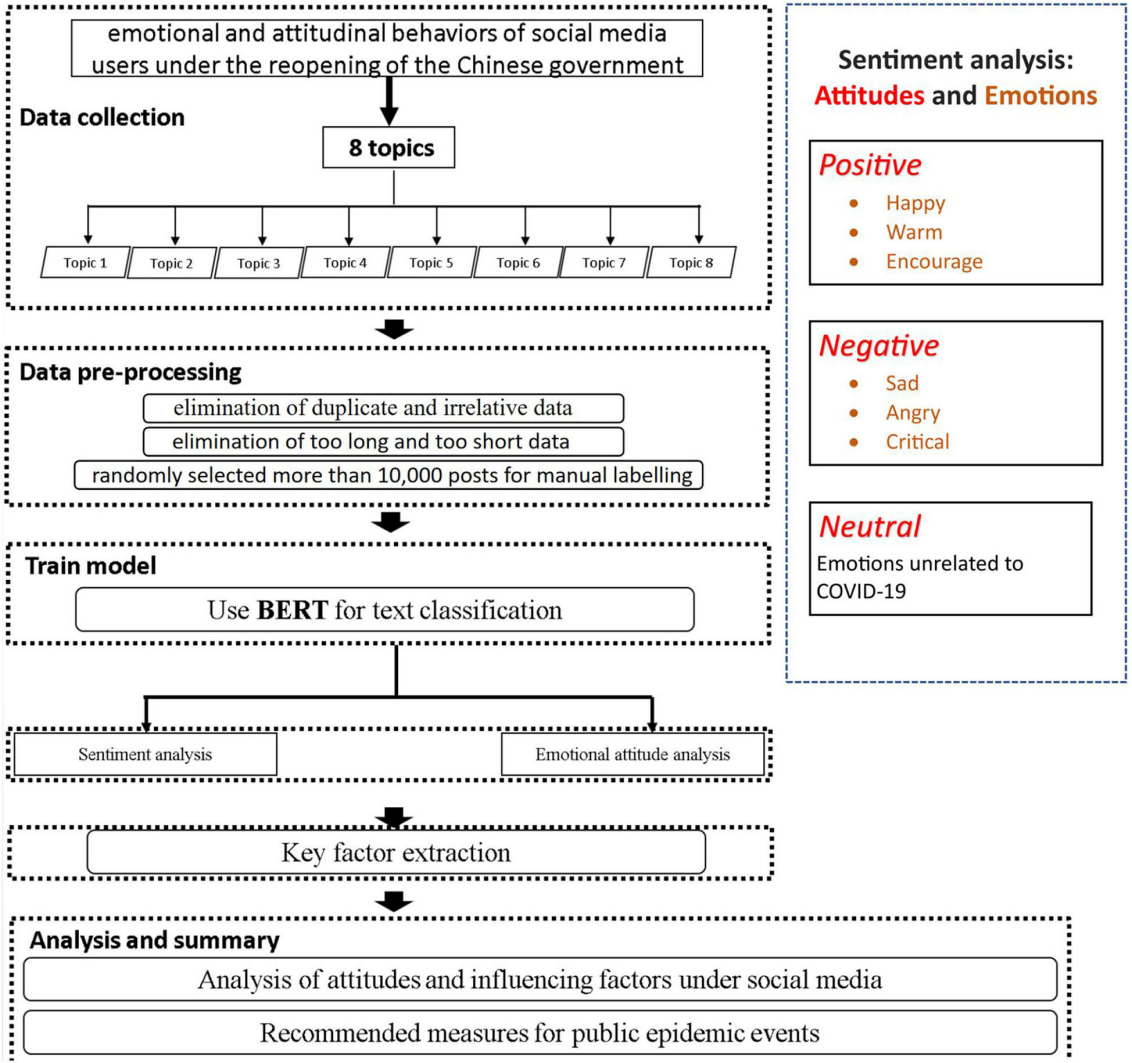
Research framework and methods of social media users' emotions and attitudes.

Further details on these steps can be found in the following sections, and a general representation is provided in Figure 1.

### 3.1. Setting

On December 7, 2022, the State Council of China issued 10 epidemic prevention policies, announcing that China's 3-year epidemic began to be fully liberalized. The announcement of the State Council marks the almost complete opening of the epidemic in China in the past 3 years, ushering in a relaxed post-epidemic era! On December 13, the trip code went offline, marking the end of the 3-year epidemic prevention policy. It triggered a heated discussion on the Internet, and the public expressed their views one after another.

### 3.2. Data collection

Weibo is a popular social media platform that enables instant information sharing, communication, and interaction among users through various mobile terminals such as PCs and mobile phones. With its open platform architecture, Weibo provides a simple and unprecedented way for users to publish content in realtime to the public. It has transformed the way information is transmitted on the internet and facilitated the instant sharing of information. According to Weibo's financial report for the fourth quarter and the whole year of 2020 (2022?), the platform had 521 million monthly active users and an average of 225 million daily active users in December 2020 (2022?). On Weibo, users can express their opinions on different themes, which are identified by using a “#” symbol before and after the theme name. For example, themes like #We are officially moving toward the end of the epidemic life# allow users to engage in discussions related to specific topics. In our study, we analyzed the real-time hot search list on Sina Weibo and selected eight themes related to the epidemic with high discussion rates for data collection. Using Python technology, we collected a total of 125,686 online comments from December 1, 2022, to December 20, 2022 (as shown in Table 1). Each comment includes various attributes such as ID, BID, user ID, user nickname, Weibo text, headline article URL, publishing location, mentions (user), topic, reposts, comments, likes, publishing time, publishing tool, Weibo picture URL, Weibo video URL, and retweet ID.

**Table 1:** Characteristics of selected Weibo topics on China's opening up after the government announcement.

**No**.	**Topic(#...#)**	**Date**	**Comments**	**Likes**
1	Twenty measures to optimize prevention and control work	2022-11-10	21,593	63,984
2	Close contacts who no longer judge close contacts	2022-11-11	15,042	62,744
3	Criteria for delimitation and removal of epidemic risk areas	2022-11-21	10,080	32,253
4	Do not check the health code except for special places such as schools	2022-12-07	17,587	56,101
5	Farewell to health code	2022-12-08	13,073	24,870
6	Notice on Further Optimizing Epidemic Prevention and Control	2022-12-09	15,817	45,970
7	The new ten items of epidemic situation	2022-12-10	19,123	31,669
8	It is expected to reach the peak of infection within 1 month	2022-12-11	13,371	27,373

### 3.3. Data preprocessing

**Figure 2:**
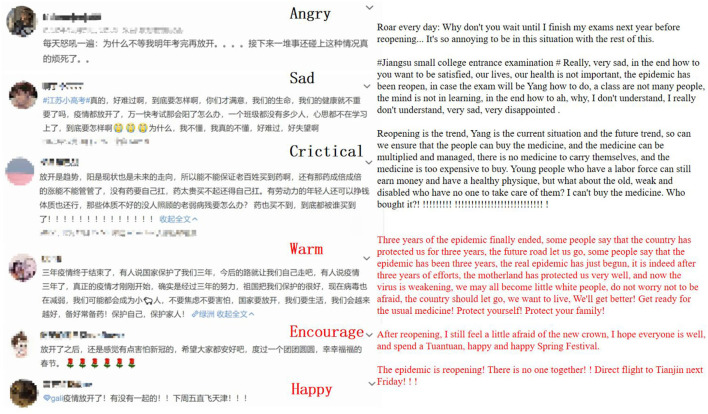
Example of performed data labeling.

To ensure data quality, we performed data cleaning and sorting on the obtained dataset. We retained only the text of the microblogs, excluding posts with fewer than 10 or more than 100 words, as well as removing duplicates. Additionally, for improved machine learning in sentiment analysis, we randomly selected over 10,000 posts related to the “release epidemic control” policy for manual labeling. Regarding the attitude reflected in each post, we utilized three labels: “negative,” “positive,” and “neutral.” Posts unrelated to the epidemic were marked as “neutral.” To capture the emotions expressed in the posts, we further subdivided the “positive” and “negative” attitudes into three types of emotions each. Specifically, the “positive” attitude encompassed emotions such as “happy,” “warm,” and “encouraging,” while the “negative” attitude included emotions like “sad,” “angry,” and “critical.” An example of the annotated data is shown in Figure 2.

### 3.4. Segmentation

Segmentation plays a crucial role in natural language processing as it transforms unstructured textual data into structured data, establishing a standardized representation. This process involves breaking down the text into smaller units, such as words or tokens, enabling more granular analysis. By segmenting and structuring the text data, it becomes easier to perform various analyses, including text classification and text analysis. In our text classification model, we employ the trained Chinese word segmentation provided by RoBERTa as our segmentation tool. This allows us to leverage the capabilities of RoBERTa in segmenting Chinese text effectively. Additionally, for constructing the word cloud map using Sina Weibo data, we utilize the jieba thesaurus. Jieba is an excellent third-party Chinese word segmentation library in Python, utilizing a Chinese thesaurus to calculate association probabilities between Chinese characters. This enables the formation of phrases with high association probabilities. The combination of RoBERTa and jieba enables us to effectively segment the Chinese text, facilitating subsequent analyses and providing valuable insights.

### 3.5. Text classification

**Figure 3:**
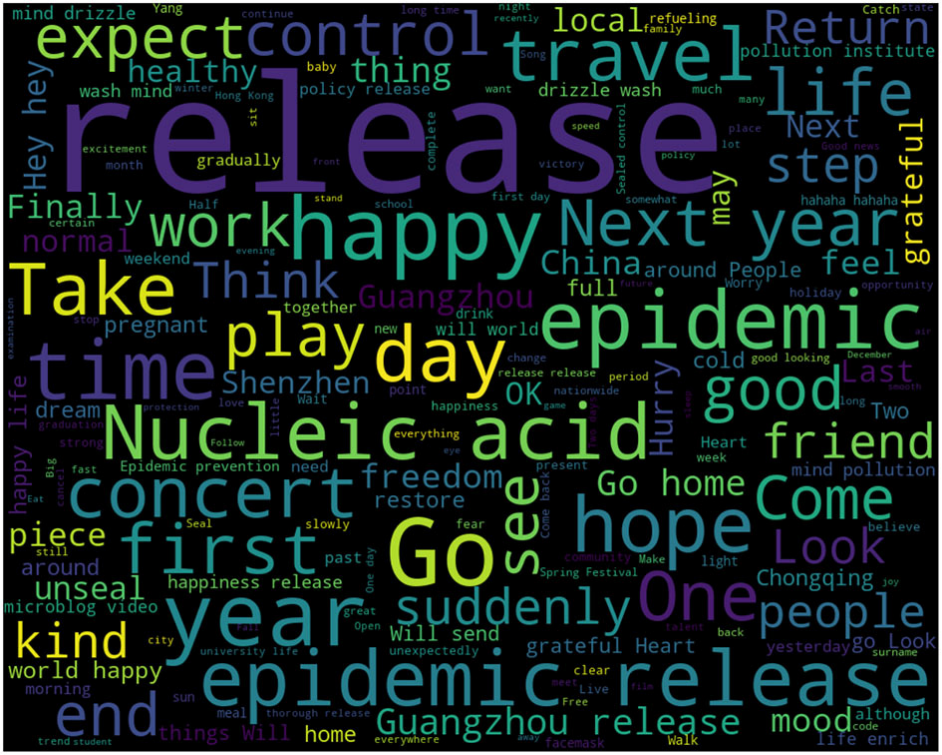
Training method of model.

We employ text classification as a method to determine the attitudes and emotions expressed by the public in each post. Text classification involves automatically categorizing text data according to predefined classification rules or standards. It typically consists of two steps: building the feature representation of the text and training the classification model. In this study, we adopt the widely used pre-training+fine-tuning method in the field of natural language processing. Pre-training involves training the model on a large corpus of unlabeled text data, using two specific tasks: Next Sentence Prediction (NSP) and Masked Language Modeling (Mask LM). In the NSP task, the model is trained to predict whether the second sentence follows the first sentence in the original text, enabling it to capture sentence-level relationships and understand semantics. In the Mask LM task, certain words in each sentence are randomly masked, and the model predicts these hidden words based on the context provided by the remaining words. By comparing the predicted results with the original text, the model learns to fill in the masked words effectively. After completing the pre-training stage, the model can be fine-tuned to adapt to specific characteristics and requirements. This process involves using a small portion of annotated data from the target task domain, such as Multi-Genre Natural Language Inference (MNLI), Named Entity Recognition (NER), and Stanford Question Answering Dataset (SQuAD), to achieve optimal performance. MNLI assesses the model's ability to determine the relationship between two sentences (entailment, contradiction, or neutral), while NER focuses on identifying and classifying named entities (e.g., people, organizations, locations) in text. SQuAD evaluates the model's ability to generate precise answers to questions based on given passages of text. Prominent examples of this method include Google's BERT algorithm (52) and OPENAI's GPT series (53–55). Considering the advantages of BERT and its variants over other models, as discussed by (56), we utilize the BERT-based enhancement algorithm RoBERTA (57). RoBERTA utilizes longer training times, larger batch sizes, and more data to achieve improved training results. The training method of the model is illustrated in Figure 3, where the model is first pre-trained and then fine-tuned in the downstream task. We separately trained the attitude analysis model and the emotion analysis model using their respective attitude and emotion labels.

### 3.6. Text analysis: factor extraction

**Figure 4:**
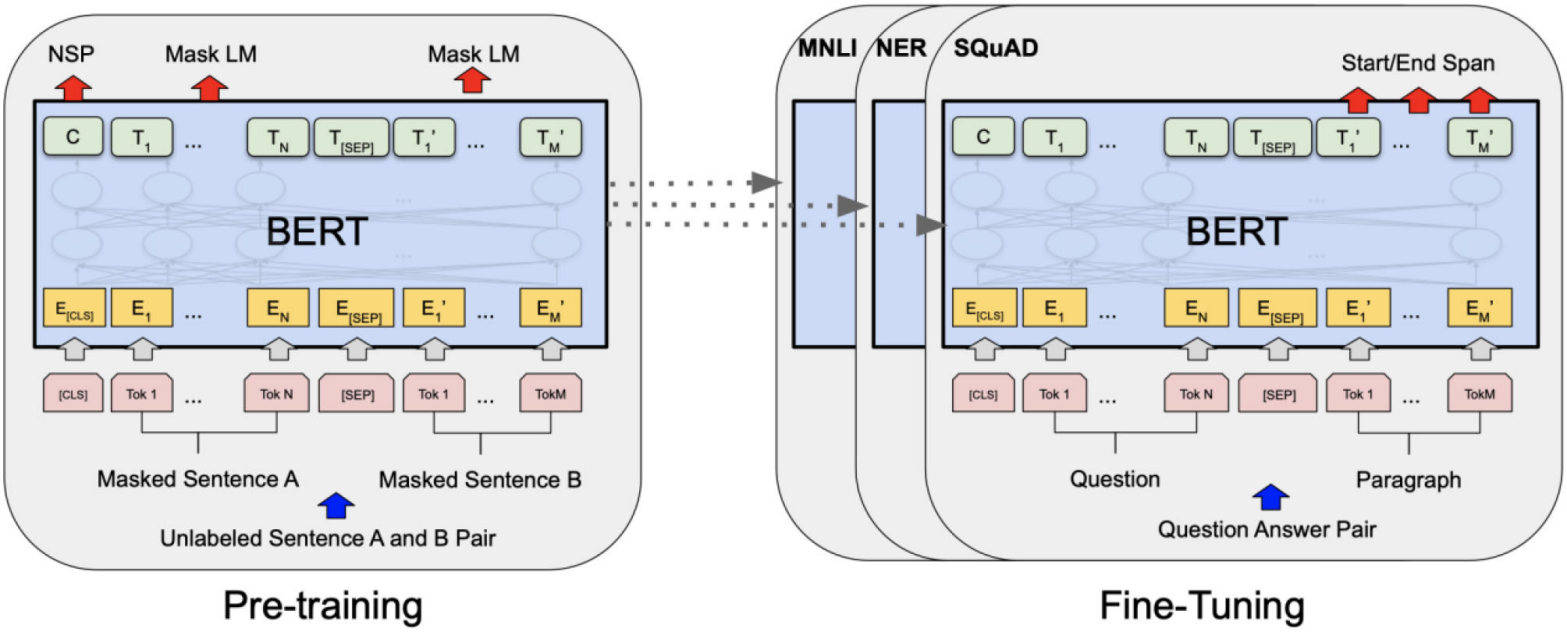
Example of word cloud.

**Figure 5:**
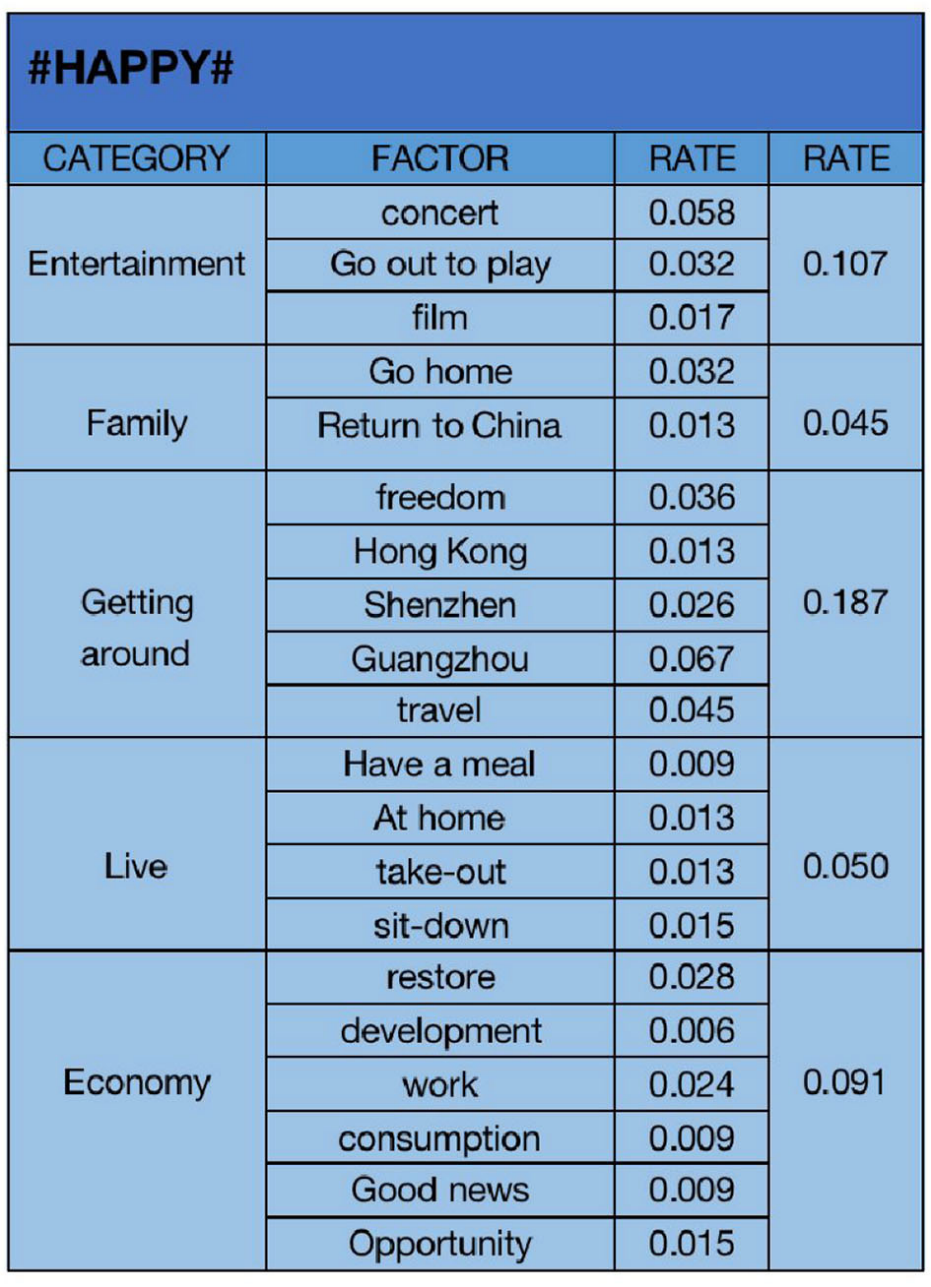
Data analysis results of “happy” emotion.

**Figure 6:**
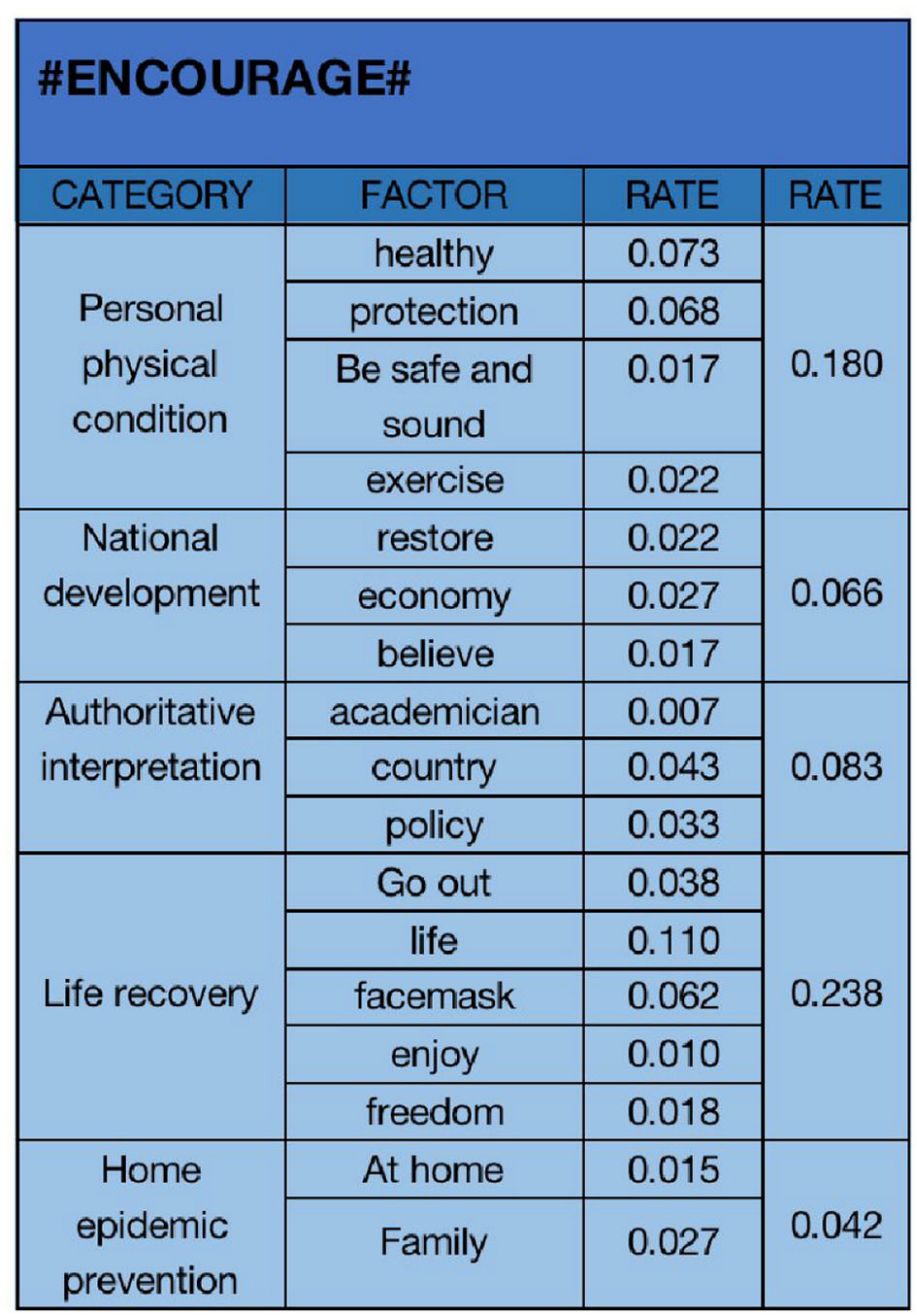
Data analysis results of “encourage” emotion.

**Figure 7:**
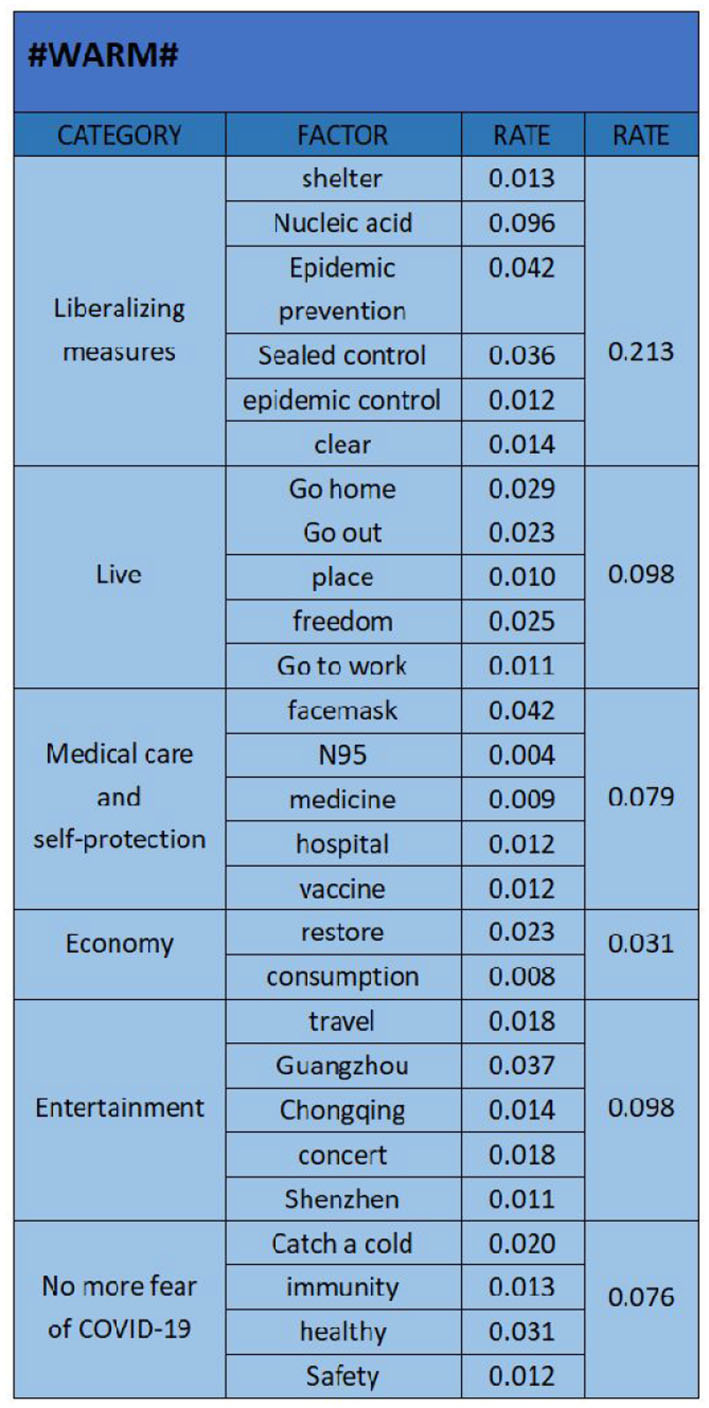
Data analysis results of “warm” emotion.

**Figure 8:**
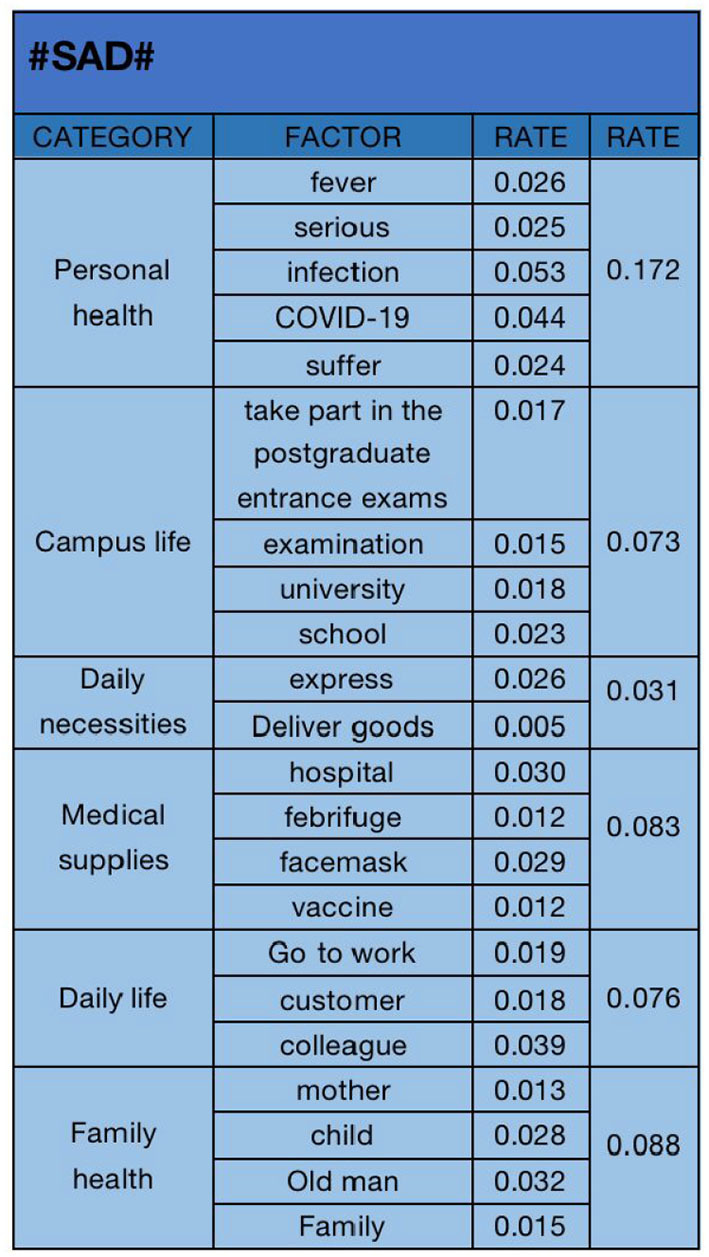
Data analysis results of “sad” emotion.

**Figure 9:**
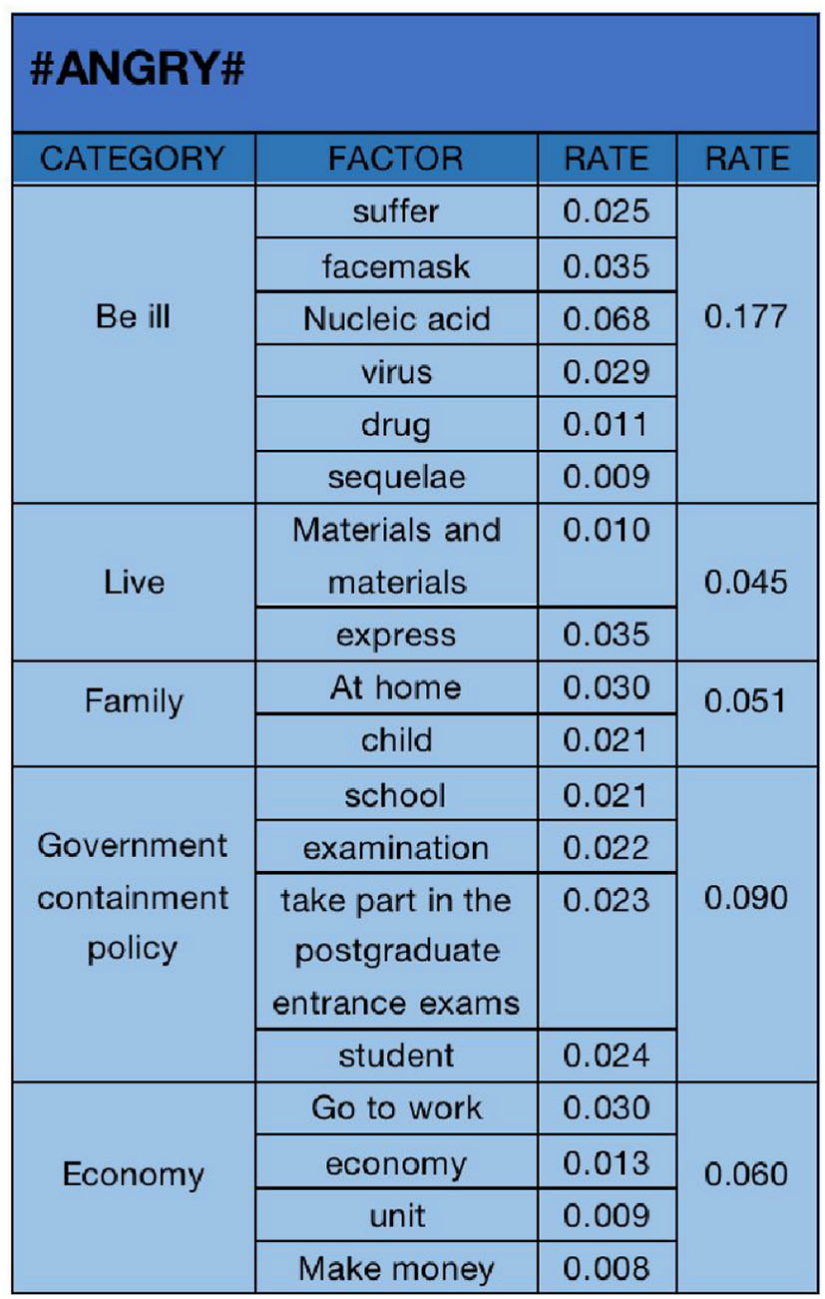
Data analysis results of “angry” emotion.

**Figure 10:**
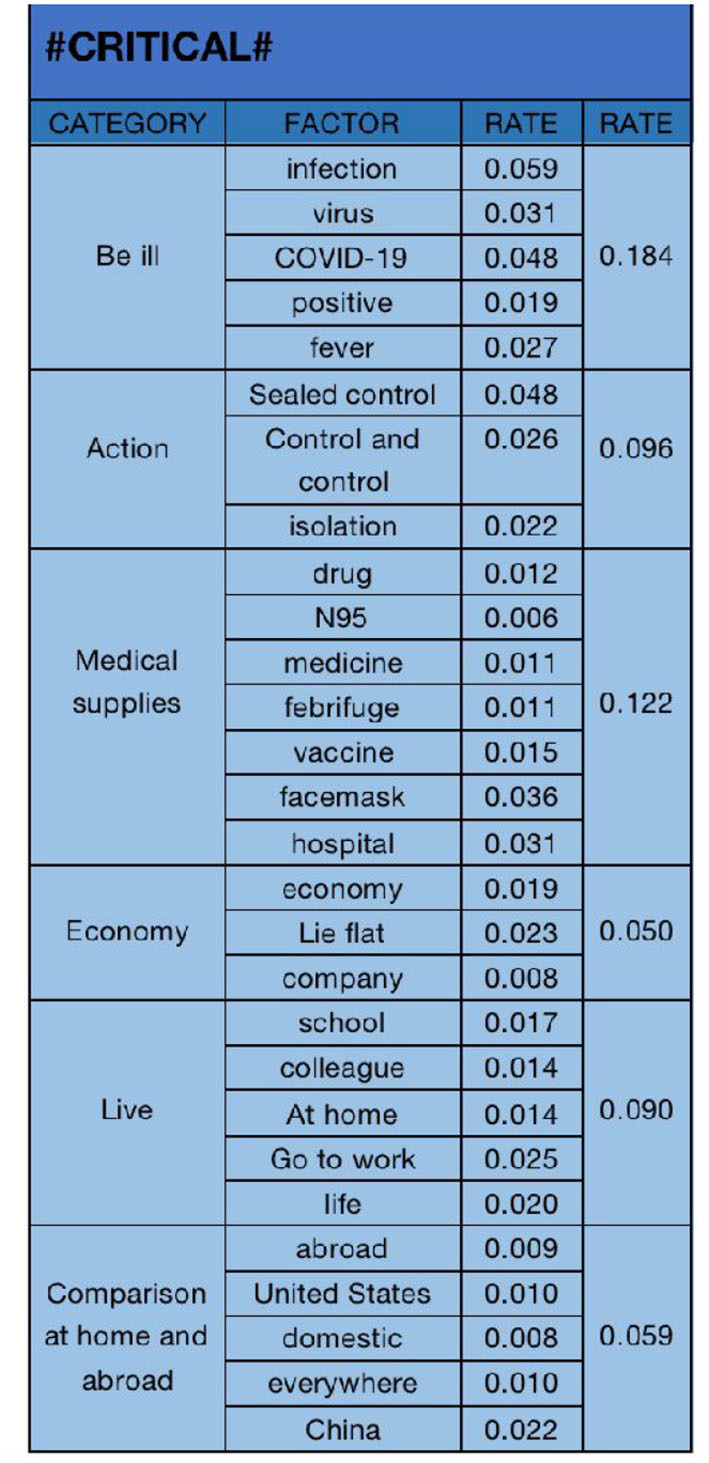
Data analysis results of “critical” emotion.

Once we have trained the attitude analysis model and the emotion analysis model, we can proceed with classifying all the data. As the classification of positive and negative attitudes encompasses three distinct emotions, we adopt a method that involves analyzing each emotion separately and then summarizing the results. For the data classified by the models, we summarize the information associated with each specific emotion. Subsequently, we perform text analysis on the collected data, generating word cloud maps that 8 represent the corresponding attitudes and emotions. From these word cloud maps, we extract the words with higher frequencies, enabling us to identify the main influencing factors for each sentiment (see Figure 4). By calculating the proportion of each factor within the original data, we can summarize the key influencing factors that contribute to a particular attitude or emotion. These factors are further classified into categories (refer to Figures 5–**10**), providing a comprehensive understanding of the underlying factors associated with different emotional responses.

## 4. Results and discussion

### 4.1. Positive attitude analysis

Positive attitude includes three emotions: “happy”, “encourage”, and “warm”. We analyze the influencing factors of the three emotions respectively, and then summarize the influencing factors of positive attitude.

#### 4.1.1. “Happy” emotional analysis

We summarize all the data classified as “happy” emotions, conduct text analysis to get the key influencing factors, and then count the proportion of each key factor in all the “happy” data, as shown in Figure 5. In the text analysis of “happy” emotion data, we select “concert (0.058)”, “go out to play (0.032)”, “film (0.017)”, “go home (0.032)”, “return to China (0.013)”, “freedom (0.036)”, “Hong Kong (0.013)”, “Shenzhen (0.026)”, “Guangzhou (0.067)”, “travel (0.045)”, “have a meal (0.009)”, “at home (0.013)”, “take-out (0.013)”, “sit-down (0.015)”, “restore (0.028)”, “development (0.006)”, “work (0.024)”, “consumption (0.009)”, “good news (0.009)”, and “opportunity (0.015)”, and according to the characteristics of these factors, they are classified into five categories. “Entertainment (0.107)”, “family (0.045)”, “getting around (0.187)”, “live (0.050)”, and “economy (0.091)”, “Entertainment” is reflected in the public's expression of the various entertainment activities they want to carry out after the epidemic was released. Compared with the limited entertainment activities during the previous epidemic control, this change makes them happy. “Family” reflect that people who leave home to work and study after the epidemic situation is released can finally feel happy to return to their families without considering being isolated. “Getting around” is reflected in the fact that people can travel freely in various cities without checking nucleic acid and travel codes after the epidemic situation is released. “Life” reflects that people's life finally returns to normal, they can freely order take-out food or go out to eat, and their life gradually returns to the state before the epidemic. The economy is reflected in the fact that after the epidemic is closed and controlled, those industries limited by the epidemic finally have the opportunity to develop, and the economic situation of the country will gradually improve so that people can find their desired jobs. The proportion of “travel” is the highest. Under the policy of opening up the epidemic, the public can travel freely, which is the primary factor that makes them happy. Secondly, it is also an important factor of “happy” emotion to be able to participate in recreational activities as much as possible and the economic form will be improved.

#### 4.1.2. “Encourage” emotional analysis

In the same way, we also conduct text analysis on all the data of encouraging emotion. As shown in Figure 6, we choose “healthy (0.073)”, “protection (0.068)”, “Be safe and sound (0.017)”, “exercise (0.022)”, “restore (0.022)”, “economy (0.027)”, “believe (0.017)”, “academician (0.007)”, “country (0.043)”, “policy (0.033)”, “go out (0.038)”, “life (0.110)”, “facemask (0.062)”, “enjoy (0.010)”, “freedom (0.018)”, “at home (0.015)”, “family (0.027)”. The 18 key factors of “happy” are classified into five categories: “personal physical condition (0.180)”, “national development (0.066)”, “authoritative interpretation (0.083)”, “life recovery (0.238)”, and “home epidemic prevention (0.042)”. “Authoritative interpretation” reflects that the public encourages the liberalization of the COVID-19 because the authoritative interpretation of the country and academicians shows that the liberalization of the epidemic is not a choice of lying flat, but has been overcome. From it, we can see that the proportion of “returning to life before the epidemic” is the highest. After the epidemic opens, life will slowly return to life before the epidemic. This is the primary factor of “encourage” emotion, and “personal physical condition” is also an important factor of “encourage” emotion.

#### 4.1.3. “Warm” emotional analysis

The results of “warm” emotional data text analysis are shown in Figure 7. We have selected “shelter (0.013)”, “nucleic acid (0.096)”, “epidemic prevention (0.042)”, “sealed control (0.036)”, “epidemic prevention and control (0.012)”, “clear (0.014)”, “go home (0.029)”, “go out (0.023)”, “place (0.010)”, “freedom (0.025)”, “go to work (0.011)”, “facemask (0.025)”, “n95 (0.004)”, “medicine (0.009)”, “hospital (0.012)”, “vaccine (0.012)”, “restore (0.023)”, “consumption (0.008)”, “travel (0.018)”, “Guangzhou (0.037)”, “Chongqing (0.014)”, “concert (0.018)”, “Shenzhen (0.011)”, “Catch a cold (0.020)”, “immunity (0.013)”, “healthy (0.031)”, “safety (0.012)”. The 27 key factors are classified into six categories: “liberalizing measures (0.213)”, “live (0.098)”, “medical care and self-protection (0.079)”, “economy (0.031)”, “entertainment (0.098)”, and “no more fear of COVID-19 (0.076)”. “Liberalizing measures” is reflected in the improvement of previous measures related to epidemic control after the epidemic control. “live” is reflected in that people's encouraged be restored to before the epidemic, and everyone can travel and live freely. “Medical care and self-protection” is reflected in the fact that people can freely purchase relevant medical supplies after the epidemic situation is released, and there is no need to buy cold medicine as before. “No more fear of COVID-19” is reflected in people's understanding that the current COVID-19 is just a serious cold, no longer afraid of COVID-19, more passionate embrace of life. It can be seen that “measures related to the relaxation of the epidemic” is the primary factor that leads to the public's “enthusiasm”. Under the open state of the epidemic, restrictions such as nucleic acid testing and travel codes are imposed on people, which is why the public supports the opening up of the epidemic with enthusiasm. Secondly, “life” and “entertainment” are also important influencing factors. In the open state of the epidemic situation, life will slowly recover, People can also engage in their favorite entertainment activities.

### 4.2. Negative attitude analysis

Negative attitudes include three emotions: “sad”, “angry”, and “critical”. We analyze the influencing factors of the three emotions respectively and then summarize the influencing factors of negative attitudes.

#### 4.2.1. “Sad” emotional analysis

The text analysis results of “sad” emotional data are shown in Figure 8. We selected “fever (0.026)”, “serious (0.025)”, “infection (0.053)”, “COVID-19 (0.044)”, “suffer (0.024)”, “take part in the postgraduate entrance examination (0.017)”, “examination (0.015)”, “university (0.018)”, “school (0.023)”, “express (0.026)”, “deliver goods (0.005)”, “hospital (0.030)”, “febrifuge (0.012)”, “facemask (0.029)”, “vaccine (0.012)”, “go to work (0.019)”, “customer (0.018)”, “colleague (0.039)”, “mother (0.013)”, “child (0.028)”, “old man (0.032)”, “family (0.015)”. The 22 key factors of are classified into six categories: “personal health (0.172)”, “campus life (0.073)”, “daily necessities (0.031)”, “medical supplies (0.083)”, “daily life (0.076)”, and “family health (0.088)”. We can see that “personal health” is the primary factor leading to “sad” emotion, and “family health” and “medical supplies” are also important factors. In the open state of the epidemic, most people are infected, and their illness makes them feel sad, and many people can not buy medical supplies.

#### 4.2.2. “Angry” emotional analysis

The text analysis results of “Angry” emotional data are shown in Figure 9. We selected 18 key factors, including “suffer (0.025)”, “facemask (0.035)”, “nucleic acid (0.068)”, “virus (0.029)”, “drug (0.011)”, “sequelae (0.009)”, “materials (0.010)”, “express (0.035)”, “at home (0.030)”, “children (0.021)”, “school (0.021)”, “examination (0.022)”, “take part in the postgraduate entrance examination (0.023)”, “student (0.024)”, “go to work (0.030)”, “economy (0.013)”, “unit (0.009)”, and “make money (0.008)”, They are classified into five categories: “be ill (0.177)”, “live (0.045)”, “family (0.051)”, “government containment policy (0.090)”, and “economy(0.060)”. It can be seen that “be ill” is the primary factor leading to “angry” emotion. In the open state of the epidemic, most people are infected, resulting in their anger at the open policy. In addition, the “government containment policy” is also an important factor in the “angry” emotion. Many students are angry that the epidemic is open before the postgraduate entrance examination, which affects their examination and postgraduate entrance examination status.

#### 4.2.3. “Critical” emotional analysis

The text analysis results of “critical” emotional data are shown in Figure 10. We have selected “infection (0.059)”, “virus (0.031)”, “COVID-19 (0.048)”, “positive (0.019)”, “fever (0.027)”, “sealed control (0.048)”, “control (0.026)”, “isolation (0.022)”, “drugs (0.012)”, “N95 (0.006)”, “medicine (0.011)”, “febrifuge (0.011)”, “vaccine (0.015)”, “facemask (0.036)”, “hospital (0.031)”, “economy (0.019)”, “lie flat (0.023)”, “company (0.008)”, “school (0.017)”, “colleague (0.014)”, “at home (0.014)”, “go to work (0.025)”, “life(0.020)”, “abroad (0.009)”, “the United States (0.010)”, “domestic (0.008)”, “everywhere (0.010)”, and “China (0.022)”. The 28 key factors are classified into six categories: “be ill (0.184)”, “action (0.096)”, “medical supplies (0.122)”, “economy (0.050)”, “live (0.090)”, and “comparison at home and abroad(0.059)”. The “comparison at home and abroad” is reflected in the fact that after the opening of the epidemic in China, some public expressed their dissatisfaction and expressed their feelings by comparing the domestic situation with the foreign situation. It can be seen that “be ill” is also the primary factor of “critical” emotion, and “medical supplies” is also an important factor.

### 4.3. Discussion

**Figure 11:**
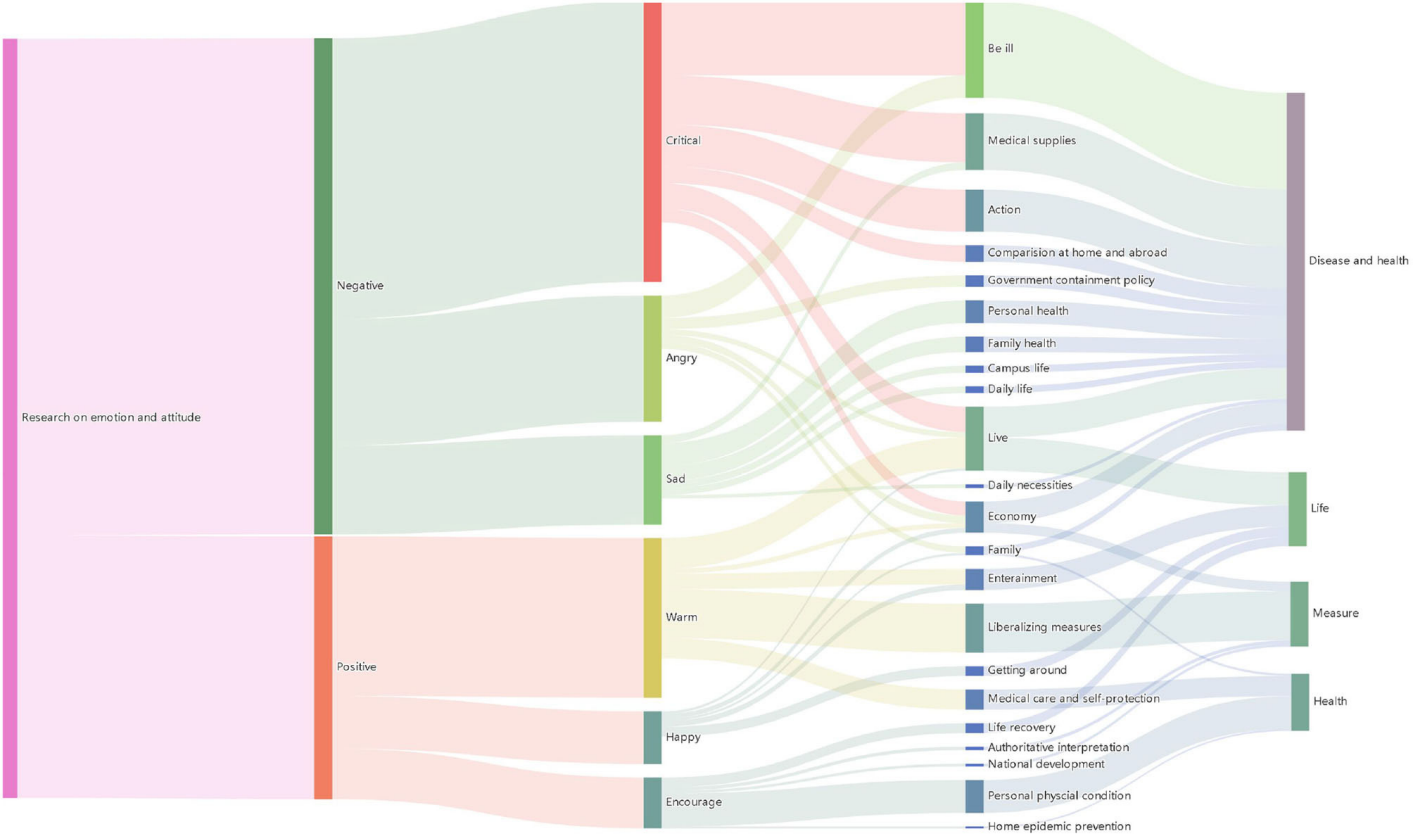
Results of the study on the emotions and attitudes of China's first opening up of epidemic control.

Currently, research uses Weibo data to discuss the emotional attitudes of social media users and their influencing factors, which are reflected in their online comments. By collecting a large amount of data and using machine learning methods, we explored and described the emotions, attitudes, and factors of social media users toward the open policy of the epidemic. The pandemic has had an impact on the public, and emotional expression is widespread on social media (58). Furthermore, we found that the emotional attitudes of social media users are closely related to their actual needs, rather than the release of policies. Although the government has issued an open epidemic policy, discussions related to the epidemic have not ended but have become a hot topic. We discuss the main findings shown in Figure 11 in detail.

#### 4.3.1. Attitudes of social media users

Previous research suggested that most users had a positive attitude toward the government's implementation of the open policy for the epidemic (58). However, we found that the majority of users had a negative attitude toward the policy release and did not want the government to open epidemic prevention and control policies (59). They expected the Chinese government to maintain the blockade policy of “zero cases” domestically and epidemic prevention and control measures for foreign countries. The reason for the negative attitude of citizens is that after the epidemic policy is opened, cases will rise sharply, and people feel uneasy. Our research further found that through 3 years of epidemic prevention and control policy implementation (60), life in China has returned to normal, cases have been basically eliminated, and most Chinese citizens have not been infected with COVID-19. The public has accepted and adapted to this way of life, and sudden policy changes have caused panic among the public. Therefore, on the surface, users in China and other regions are worried about the health crisis caused by the implementation of open policies and the rise in cases. However, the reasons behind this are different. This has led to negative attitudes in China mainly being reflected in sad, angry, and critical, which also reflects the public's dissatisfaction with policy changes. At the same time, we also analyzed positive attitudes, and found that users focused on the theme of “entertainment life” after the transformation of epidemic prevention and control policies in positive emotions. Therefore, the main emotions included happy, encourage, and support.

#### 4.3.2. Emotions of social media users

Positive attitudes, users responded positively to the government's open policy. Despite the problems caused by the epidemic policy, people actively adjusted their attitudes (61), accepted the relevant policies, and enjoyed spending time with their families. This study found that during the 3 years of implementation of China's epidemic prevention and control policy, the public's daily travel was restricted, life was inconvenient, and the economy was impacted. The sudden change in epidemic policy brought hope to the public. They are more eager to return to normal life before the epidemic (41). Therefore, positive attitudes are mainly reflected in three emotions: happy, encourage, and support, which also reflect some public support for policy changes.

Negative attitudes, users are worried about health issues and question the government's open policy (62). In previous studies, due to the impact of the epidemic, the economy declined, unemployment rates rose, people's income decreased, and a lot of negative emotions were generated. The government's open epidemic policy is to ease social conflicts and increase economic income. From this perspective, policy changes will have a positive impact on the public's lives. This study found that the majority of the public are dissatisfied with the sudden opening of the epidemic policy, and they prefer to maintain the current living status. They are worried that the open epidemic policy will lead to more serious consequences and hold a pessimistic emotion toward the epidemic opening. Negative attitudes are mainly reflected in three emotions: sad, angry, and critical.

#### 4.3.3. Factors influencing social media users' attitudes and emotions

In positive user attitudes, previous research has mainly discussed the overall trend of positive attitudes (63). We further studied the specific factors influencing emotions under positive attitudes, including “happy,” “encourage,” and “support.” The main influencing factors are themes such as “freedom of movement,” “return to normal life,” and “government policies.” Through data text analysis, the following conclusions were drawn:

In the “happy” positive emotion, the main influencing factors are the five categories of “entertainment,” “family,” “travel,” “life,” and “economy.” In terms of specific expressions, “concerts” are the most discussed topic in the “entertainment” category. This also reflects that during the epidemic control period, there were fewer outdoor entertainment activities, and the public hopes for a colorful social entertainment life. “Travel” is the highest proportion of influencing factors in the user's “happiness” emotion. Due to the epidemic control policies, the government has imposed various restrictions on public travel. Faced with the open policy, travel is the public's primary choice. In the “encouragement' positive emotion, the main influencing factors are the five categories of “personal physical condition,” “national development,” “authoritative interpretation,” “return to pre-epidemic life,” and “family epidemic prevention.” In terms of specific expressions, “return to pre-epidemic life” is the highest proportion of influencing factors in the public's “encouragement” positive emotion. The public's encouragement toward the open policy mainly stems from their desire for a “normal life.” In the “support” positive emotion, the main influencing factors are the six categories of “life,” “medical care,” “economy,” “entertainment,” “treat it as a cold, no longer afraid,” and “epidemic-related open measures.” Among them, “epidemic-related open measures” are the highest proportion of influencing factors in the “support” positive emotion. The corresponding measures of the open policy are an important basis for the public's acceptance of the policy. Policy release alone cannot gain public recognition. Timely and effective response measures are helpful in gaining public “support.”

In negative user attitudes, previous research has focused on analyzing the influencing factors of specific groups (64). This study found that the public's negative attitude toward the sudden opening policy during the “unexpected” epidemic mainly includes three emotions: “sad,” “angry,” and “critical.” The influencing factors are mainly themed around “physical health” and “government policies.” Through data text analysis, the following conclusions were drawn:

In the “sad” negative emotion, the six influencing factors include “personal health,” “campus life,” “daily necessities,” “medical supplies,” “daily life,” and “family health.” Among them, “personal health” is the highest proportion of influencing factors in the “sadness” negative emotion. After the epidemic control policy was opened, the public's biggest concern was the rapid spread of the epidemic, leading to illness. The public expressed concern about their own health status. In the “angry” negative emotion, the five main influencing factors are “illness,” “life,” “family,” “government control policies,” and “economy.” “Illness” is the highest proportion of influencing factors in the “angry” negative emotion. The Chinese public's biggest fear is getting “COVID-19,” afraid of dying or suffering from sequelae due to “COVID-19,” so they expressed “anger” toward the government's open policy. In the “critical” negative emotion, the six main influencing factors are “illness,” “action,” “medical supplies,” “economy,” “life,” and “domestic and foreign comparisons.” Similarly, “illness” is also the main influencing factor in the “critical” negative emotion. Compared with the reasons for “anger,” in the “critical” negative emotion, more of the public expressed criticism of the sudden changes in government policies, and in the early stages of policy implementation, the government did not have corresponding policy plans and measures, which triggered public criticism of government actions.

Based on the above analysis, we found that the emotional attitudes of social media users are mainly based on their actual lives, which also reflects the insufficient means of the Chinese government to respond to open policies. Under sudden policy changes, social media users mainly showed negative emotional attitudes. The factors influencing negative attitudes are mainly centered around the themes of “physical health” and “government policies”. After the outbreak of the epidemic, the coronavirus began to spread widely, and most people were tortured by various symptoms of the epidemic, and the supply of related medical resources was insufficient. People were plunged into an atmosphere of fear and anxiety, with the main emotions including “sad”, “angry”, and “critical”. The analysis of the factors influencing positive attitudes is mainly centered around the themes of “freedom of travel”, “returning to life”, and “government policies”. After the epidemic was controlled, policies related to epidemic control were gradually lifted, and people's lives could return to the way they were before the epidemic. They longed for freedom of travel and enjoyed various entertainment activities, with the main emotions including “happy”, “encourage”, and “support”.

## 5. Conclusion

Previous research has revealed the necessity of conducting sociological investigations into emotions to fully understand emotions and social life. The lack of emotional communication due to the impact of quarantine policies during the pandemic has resulted in negative emotions and psychological problems. However, the factors behind emotional responses are often difficult to reflect in “small sample” data collection and are more difficult to assess from a “micro” perspective, making it difficult to make judgments at the societal level. From a neuroscientific perspective, attitudes and emotions can affect cognitive responses, and severe cases can lead to the onset of other diseases. Furthermore, understanding the physiological mechanisms behind attitudes and emotions can help in the treatment and prevention of related diseases. However, the research methods and conclusions of sociology and neuroscience seem to be unsuitable for government management in the face of effective pandemic management. Therefore, this article takes the sudden implementation of China's pandemic opening policy as the research object and uses the online comments of “Weibo” hot topics as the dataset to study social media users' attitudes, emotions, and influencing factors toward the pandemic opening policy. As is shown in Figure 11, the following conclusions were drawn through content analysis.

Firstly, in terms of social media users' attitudes, they mainly hold a negative attitude toward the “sudden” pandemic opening policy, and the proportion is quite large. The proportion of positive and neutral attitudes among the public is roughly equal. This also shows that the “sudden” pandemic opening policy is a positive measure at the national level to respond to the pandemic, but the public maintains a pessimistic view of the implementation of this policy. Secondly, in terms of social media users' emotions, positive emotions such as happiness, encouragement, and warmth express appreciation for the “sudden” opening policy. Negative emotions such as sad, angry, and critical express concerns about the “sudden” opening policy. Finally, the factors that influence the above attitudes and emotions are mainly “travel,” “entertainment,” “returning to normal life,” “personal physical condition,” and “opening measures” for positive attitudes. After classifying the influencing factors in the data, we found that the themes of “freedom of travel,” “returning to normal life,” and “government policies” are the main factors that affect the public's judgment of the pandemic opening policy. “Personal health,” “family health,” “be ill,” “government quarantine policies,” and “medical supply” are the main factors that affect negative emotions. After classifying the influencing factors in the data, we found that the themes of “physical health” and “government policies” are the main factors that affect the public's choice of negative emotions.

### 5.1. Implications

In the face of such sudden changes in epidemic prevention policies, we suggest the following:

Firstly, the public generally has a negative attitude toward “sudden” measures, and the government needs to be prepared to address this negativity. When formulating relevant policies, the government hopes to gain public acceptance and support. Therefore, in the future, when releasing similar policies, the government should prepare in advance for the supply of public life, formulate detailed measures to respond, and provide relevant health consultation services. These methods can increase the public's positive emotions and help improve their goodwill and support for the government. Secondly, “life”, “health”, and “measures” are the primary considerations for the public's “positive” emotional attitudes toward “sudden” policies, and the government should increase its efforts in this regard and actively respond to public demands. “Getting sick” is the most worrying negative emotional factor for the public regarding sudden government policies. At this time, the most important thing is the treatment plan when “getting sick”, post-recovery body care, and issues related to life security caused by “getting sick”. The government should prepare relevant handling plans in advance. Thirdly, sufficient publicity for “sudden” policies is an important means, and these factors are the main content direction of policy publicity. It is necessary to use visual poster design, new media video releases, news media announcements, and other methods to publicize relevant government measures to the public. Through these means, it is more conducive to improving the public's emotions and attitudes toward government policies and accepting relevant policies.

In terms of emotion, the emotions (“happy”, “encourage”, and “warm”) in a positive attitude all express appreciation for the open policy. “Travel”, “entertainment”, “life recovery”, “personal physical condition” and “open measures” are the main influencing factors behind these emotional expressions. After classifying the influencing factors in the data, it is mainly “life”, “health”, and “measures” that affect the public's judgment on the open policy of the epidemic. The government hopes to be adopted and supported by the public when formulating relevant policies. Therefore, when issuing similar policies in the future, the government should ensure the supply of public life in advance, formulate detailed measures and response plans, and provide relevant health consultation services. These ways can increase the positive feelings of the public and help to improve the good feeling and warm support for the government.

### 5.2. Limitations and future research

There are certain limitations to this study. Firstly, the data only includes online comments on social media and has undergone text sentiment analysis, without involving other types of data such as the number of likes and shares. Combining these data with sentiment analysis of online comments can help study the spread and impact of social media users' emotional attitudes and behaviors. Secondly, although this paper analyzed users' emotional attitudes and factors toward the government's open policy, how do users' emotional attitudes change during the time of infection, illness, and recovery (usually 2–3 months)? What are the changing factors behind it? This dynamic development is also an interesting topic for research.

## Data availability statement

The datasets presented in this article are not readily available because the dataset shall be applied to the research of China's open policy, and commercial use is prohibited. Requests to access the datasets should be directed to QZ zhangqh@chnu.edu.cn.

## Author contributions

QZ and TN: research concept, design, and manuscript drafting. TN, QZ, JY, and XG: data collection and text classification model training. QZ, TN, JY, XG, and YL: statistical analysis, data interpretation, and review of important knowledge content. QZ and YL: obtain funding and research supervision. TN and XG: administrative, technical, and material support. All authors contributed to the article and approved the submitted version.
